# Knowledge, attitude and practice towards antimicrobial resistance among final-year undergraduate students in the health sciences at Addis Ababa University, Ethiopia

**DOI:** 10.1186/s40780-025-00534-2

**Published:** 2026-01-10

**Authors:** Fasil Dejene Bedada, Oumer Sada Muhammed, Beshir Bedru Nasir

**Affiliations:** Department of Pharmacology and Clinical Pharmacy School of Pharmacy, College of Health Sciences, Addis Ababa University, Churchill Avenue, P.O. Box 1176, Addis Ababa, Ethiopia

**Keywords:** Antimicrobial resistance, Knowledge, Attitude and practice, Health sciences students, Ethiopia

## Abstract

**Background:**

Globally, antimicrobial resistance (AMR) is a complex and growing public health challenge, primarily driven by the inappropriate use of antimicrobials. Promoting the rational use of these agents is the most effective strategy for preventing AMR. This can be achieved by improving the knowledge, attitudes, and practices of both prescribers and dispensers. Hence, this study will aim to assess knowledge, attitude and practice (KAP) of final year health sciences students regarding AMR, which helps to rationalize the use of antimicrobials.

**Objective:**

To assess KAP towards AMR among final year health sciences students at Addis Ababa University

**Methods:**

The research employed a cross-sectional study design, involving 230 final-year health sciences students from various programs, including medicine, pharmacy, nursing, midwifery, anesthesia, medical radiology technology, and medical laboratory sciences. Data were collected by Kobo toolbox online structured questionnaire and analyzed using SPSS version 27.

**Result:**

The findings indicated that while 74.5% of participants possessed moderate knowledge and 81.4% displayed positive attitudes, notable gaps were evident. Among the participants, 137(59.6%) mistakenly believed that antibiotics can eliminate viruses, and 166(72.2%) thought antibiotics were effective against colds and flu. Furthermore, concerning practices such as reserving antibiotics for future use 98(42.6 %) and 73(31.7%) participants responded sharing antibiotics with others. Despite these deficiencies, students exhibited a strong awareness of the consequences of AMR 217(94.7%) and acknowledged the role of overuse in fostering resistance 205(89.0%). Attitudes were predominantly positive, with 189(82.2%) advocating for increased AMR education and 192(83.5%) recognizing that improper use contributes to resistance. However, practical behaviors, such as prematurely discontinuing antibiotics 68(29.6 %) and an overall poor practice 52(22.5%) underscored the necessity for enhanced optimized training for health sciences students. Despite no significant link between gender or field of study and KAP, participants with moderate and good AMR knowledge were significantly more likely to have optimal practices than those with poor knowledge (AOR = 9.44 and 3.9, (P = 0.017 and 0.021) respectively). Similarly, a positive attitude significantly increases optimal practice chances by about 3.39 fold (P = 0.012).

**Conclusion:**

The study revealed moderate AMR knowledge and overall positive attitudes, but persistent misconceptions about antibiotic use. Moderate or good knowledge and neutral or positive attitudes were associated with more optimal practices. Suboptimal practices highlight the need for strengthening education, and institutional interventions including short term trainings.

**Supplementary Information:**

The online version contains supplementary material available at 10.1186/s40780-025-00534-2.

## Background

Antimicrobial resistance (AMR) has emerged as one of the most pressing global public health challenges of the 21st century, particularly in developing countries [1]. AMR compromises the effective treatment of a growing number of infectious diseases, resulting in prolonged illness, increased healthcare costs, and higher mortality rates [2].

According to the World Health Organization (WHO), AMR occurs when microorganisms such as bacteria, viruses, fungi, and parasites evolve to resist antimicrobial drugs, rendering treatments ineffective [3]. The inappropriate use of antimicrobials, including antibiotics, is a primary driver of AMR. Overprescribing, self-medication, and incomplete courses of treatment contribute to selection pressure, fostering the emergence and spread of resistant microorganisms [2].

The impact of AMR is particularly severe in developing countries [4]. In Ethiopia, AMR is a major public health threat, with an estimated 19,200 deaths directly attributable and 81,200 deaths associated with it in 2021 [5] The alarmingly high pooled prevalence of ESBL-producing *Enterobacteriaceae* in Ethiopia is 49%, with leading producers *Klebsiella pneumoniae* (74%), *Escherichia coli* (67%), and *Acinetobacter baumannii* (60%) posing a significant threat to public health [6]. Key contributing factors include the high prevalence of infectious diseases, irrational use of antibiotics, poor infection control practices, substandard medications, limited awareness of AMR, frequent misdiagnosis, and inadequate laboratory facilities for antimicrobial susceptibility testing [2]. WHO-led surveys indicate that most African countries demonstrate low levels of awareness regarding prudent antimicrobial use and report a high frequency of inappropriate antibiotic consumption [3].

In Ethiopia, the situation is especially concerning due to the high burden of infectious diseases and limited access to healthcare and essential medicines. In Ethiopia, antibiotic misuse is a major public health issue driven by inappropriate prescribing and widespread over-the-counter sales, a practice explicitly not allowed by the Ethiopian Food and Drug Authority (EFDA) [7]. Studies revealed the severity of the problem, with 48.3% of the population self-antibiotics medicating [7] and 67.7% of community pharmacists dispensing antibiotics without a prescription [8].

Health sciences students, as future healthcare providers, play a crucial role in combating AMR by raising public awareness and promoting education on the rational use of antibiotics to prevent resistance [3]. To effectively fulfill these responsibilities, undergraduate health sciences students must receive comprehensive education in microbiology, pharmacology, infectious diseases, and antibiotic stewardship [9]. Additionally, they should be trained to manage patient requests for antibiotics responsibly [5]. Ensuring adequate knowledge of antibiotic use and AMR prevention is vital in curbing resistance [10]. Health sciences students represent a pivotal group capable of influencing appropriate antibiotic use and attitudes, ultimately improving patient care and healthcare outcomes [11].

Studies have highlighted that improper antimicrobial use is common among university students, often due to a lack of adequate knowledge about antimicrobials and a high prevalence of self-medication [12]. Despite WHO recommendations advocating for antimicrobial stewardship training in undergraduate medical curricula, prior studies have primarily focused on medical students, neglecting other health sciences students who also play a crucial role in AMR prevention [13, 14]. For instance, a study assessing the knowledge, attitude and practice (KAP) of 323 paramedical students at Gondar University found that 55% had poor knowledge of antimicrobial resistance [13].

KAP surveys serve as an essential tool for evaluating public understanding and behavior regarding health-related issues. The WHO defines KAP surveys as structured methods to assess what people know, believe, and do regarding a specific topic [15]. Knowledge is examined to determine its alignment with biomedical principles, while attitudes reflect predispositions toward specific health practices [15]. Understanding these dimensions is crucial for designing effective interventions to improve antimicrobial use among newly graduating healthcare professionals.

Assessing KAP regarding antibiotic use and AMR among health sciences students can identify knowledge gaps and behavioral barriers. These insights can guide targeted interventions to mitigate AMR and promote rational antimicrobial use [4]. Although studies at other Ethiopian institutions, such as the University of Gondar, have assessed the KAP of health sciences students regarding AMR, a comprehensive investigation is notably absent at Ethiopia’s largest university, Addis Ababa University. Thus, this study aims to assess KAP towards antimicrobial resistance among final year health sciences students at Addis Ababa University, Ethiopia.

## Method

### Study design

Institutional based cross-sectional study design was employed among health sciences students (medicine, pharmacy, nursing, medical laboratory sciences, medical radiology technology, anesthesia and midwifery).

### Study area

The study was conducted at Addis Ababa University, located in the country’s capital, Addis Ababa. It was established in 1950, is the oldest and largest university in Ethiopia. It offers a wide range of undergraduate and postgraduate programs across various fields, including natural sciences, engineering, social sciences, humanities, health sciences, business, and law. College of Health Science offers programs in medicine, nursing, public health, pharmacy, and other health-related disciplines. In the 2025 academic year, there are 525 final year under graduate students including medicine, pharmacy, nursing, medical laboratory sciences, medical radiology technology, anesthesia and midwifery.

### Sample size determination

The sample size was determined using the single population proportion formula, assuming a 95% confidence level, a 5% margin of error, and a 50% proportion to maximize statistical power, which yielded an initial size of 385. Since the total population was small (*N* = 525), the Finite Population Correction formula was applied to prevent oversampling, adjusting the sample to 222. To compensate for potential non-responses and incomplete data, a 10% contingency was then added, resulting in a final target sample size of 243 participants.

### Sampling procedure and techniques

A multi-stage sampling technique was utilized. The population of 525 health sciences students was first stratified by department. From this, a sample of 243 was proportionally allocated to each stratum based on its size. For instance, the Pharmacy department (13.8% of the population) was assigned a quota of 34 students, while the Medicine department (51.4%) was assigned 125. This proportional method was applied across all departments. Finally, convenience sampling was used within each department to select available students until the pre-determined quotas were fulfilled.

### Data collection procedure and tools

The data was collected by kobo toolbox using self-administered questionnaire. The questionnaire was developed after reviewing literature of similar studies conducted in different regions of the world and the WHO questionnaire for antibiotic resistance (appendix-I) [13, 16–20]. Moreover, the questionnaires’ content, design, relevance, readability, and comprehension was then examined and evaluated by subject-matter experts. The questionnaire consisted of 28 items (3 demographic, 9 knowledge, 8 attitude and 8 practice questions).

### Data quality control measures

Prior to the actual study, a pretest of the questionnaire was done on 5% of the total sample size at Menilik II Medical and Health Science College. Prior to the actual data collection and necessary modification was made accordingly. A strict supervision was undertaken by the principal investigator and supervisors throughout the data collection period to guide and correct any ambiguity occurred during the data collection process.

### Data analysis

The completed questionnaires were checked for completeness and consistency. The collected and cleaned data was exported to SPSS version 27 for analysis. Descriptive statistics such as frequency, percentage, mean, standard deviation (SD), were done and results were presented using tables, graphs and narrative descriptions. Multivariable binary logistic regression analysis was used to assess the factors associated with participants’ practice and a univariable analysis (*p* < 0.2) to control confounders and *p* value < 0.05 was considered as statistically significant.

### Operational definitions

**Knowledge** was classified based on a modified Bloom’s cut-off: Good: 80–100% (17–21 points), Moderate: 50–79% (11–16 points) and Poor: below 50% (< 11 points).

**Attitude**: Categorized using Bloom’s original cut-off: Positive: 80–100% (24–19 points), Neutral: 60–79% (18–14 points) and Negative: below 60% (< 14 points).

**Practice**: Also classified using Bloom’s original cut-off: Good: 80–100% (24–19 points), Neutral: 60–79% (18–14 points) and Poor: below 60% (< 14 points) [13].

## Results

### Demographic characteristics of study participants

A total of 230 participants were included in the study with a response rate of 94.7%. The respondents had a mean age of 23.45 ± 1.60 years, with ages ranging from 20 to 27 years. In terms of field of study, the majority were from Medicine (39.8%), while the fewest participants were from Midwifery 9(4.8%) (Table 1).

**Table 1:** Socio demographic characteristics of study participants

Variables		Frequency	Percent (%)
Sex	Female	120	52.4
	Male	110	47.6
	Total	230	100
Department	Medicine	120	39.8
	Pharmacy	34	21.2
	Nursing	18	9.1
	Midwifery	9	4.8
	Medical Laboratory	14	6.1
	Anesthesia	17	10.8
	Medical Radiology technology	18	7.4
	Total	230	100

### Participants’ knowledge towards antimicrobial resistance

**Fig. 1:**
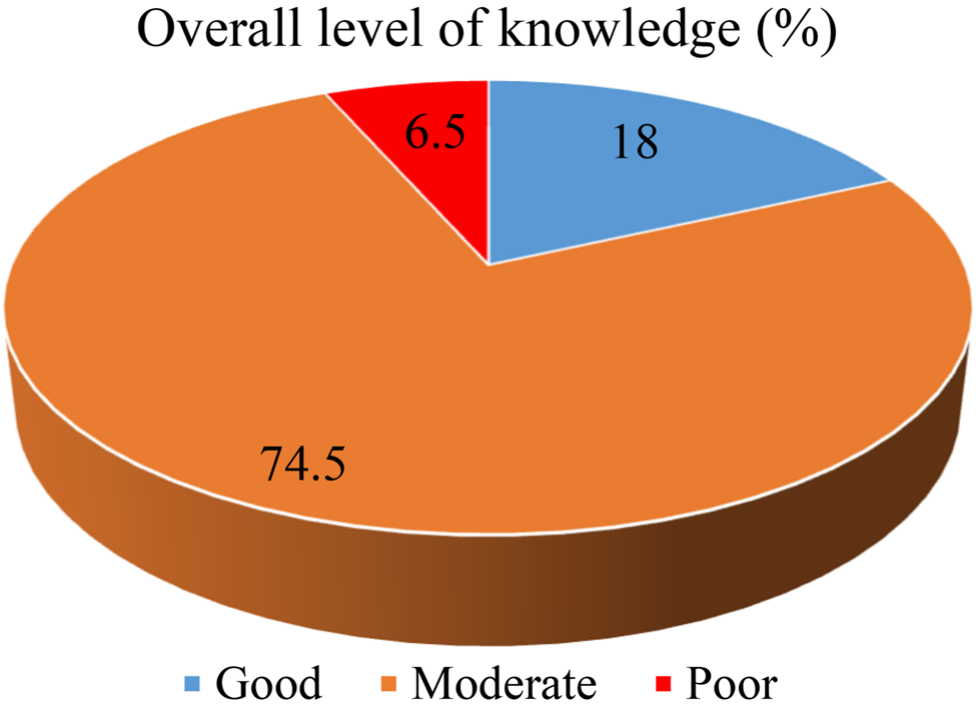
Participants’ over knowledge about AMR

Among the study participant, 171(74.5%) had a moderate level of knowledge while (6.5%) had poor knowledge (Fig. 1).

The majority 165 (71.7) knew that antibiotic usage disturbs the gut flora and causes diarrhea and super-infection. Of 230 participants, 177 (76.9%) of them conceived that frequent use of antibiotics would decrease drug efficacy. More than 218(94.7%) of the study participants knew AMR and its consequence. However, the majority of participants answered incorrectly to questions regarding the importance of antibiotics for the common cold and flu 166 (72.2%), and the belief that antibiotics are powerful medicines that can kill both viruses and bacteria 137(59.6%). Poor patient counseling 203(88%) and prescribers’ poor knowledge 185(80.4%) were seen as key drivers of misuse, alongside self-medication 177(76.7%). Overuse consequences like AMR 205(89.1%) and adverse reactions191 (83%) were well-recognized and clinical condition 220(95.5%) heavily influenced prescribing, unlike microbiological results 113(48.8%). Lack of diagnostic tools (100%) and poor hygiene 211(91.7%) were top AMR causes, though self-medication’s role was under recognized 44(18.6%). Solutions included consulting experts 177(76.9%) and resistance profiling 161(70%), but only 6(2.6%) emphasized changing prescriber/patient attitudes (Table 2).

**Table 2:** Participants’ knowledge about AMR among final year health sciences students at addis Ababa University, 2025

		Correct *N*(%)	Incorrect correct *N*(%)
1. Antibiotic usage disturbs the gut flora and causes diarrhea and super-infection		165 (71.7)	65 (28.3)
2. Are antibiotics powerful medicines to kill virus and bacteria		93 (40.4)	137 (59.6)
3. Does frequent use of antibiotics decrease the efficacy of treatment?		177 (76.9)	53 (23.1)
4. Do antibiotics speed up the recovery of common cold, cough and flu?		64 (27.8)	166 (72.2)
5. Have you ever heard about antimicrobial resistance and its consequence		218 (94.7)	12 (5.3)
6. Which of these do you think may promote the inappropriate use of antimicrobials	Poor counseling of patients	203 (88.2)	27 (11.8)
	Poor skills and knowledge of prescribers	185 (80.4)	45 (19.6)
	Patient self-medication	177 (76.9)	53 (23.1)
	Inadequate supervision	124 (53.9)	106 (46.1)
7. Which of the following do you think are the consequences of antimicrobials overuse	Antimicrobial resistance	205 (89.1)	25 (20.9)
	Adverse drug reactions and medication errors	191 (83)	39 (27)
8. Which of these factors may influence the decision to start antimicrobial therapy	Patients clinical condition	220 (95.6)	10 (4.5)
	Positive microbiological results in symptomatic patients	112 (48.7)	118 (51.3)
9. Which of the following promote antimicrobial resistances	Inappropriate prescribing habits of antibiotics	190 (82.6)	40 (17.4)
	Lack of effective diagnostics tools to diagnose bacterial infections	230 (100)	0 (0)
	Patients self-medication with antibiotics without consulting health professionals	43 (18.7)	187 (81.3)
	Spread of bacteria in healthcare settings due to poor hygiene practices	211 (91.7)	19 (8.3)
10. Which of the following do may help to control antimicrobial resistance?	Consulting with infectious diseases experts	177 (76.9)	53 (23.1)
	Obtaining local antibiotic resistance profile	161 (70)	69 (30)
	Targeting antimicrobial therapy to likely pathogens	171 (74.3)	59 (25.7)
	Changing the attitudes of prescribers and patients to reduce unnecessary antibiotic usage	6 (2.6)	224 (97.4)

### Study participants’ attitude towards antimicrobial resistance

**Fig. 2:**
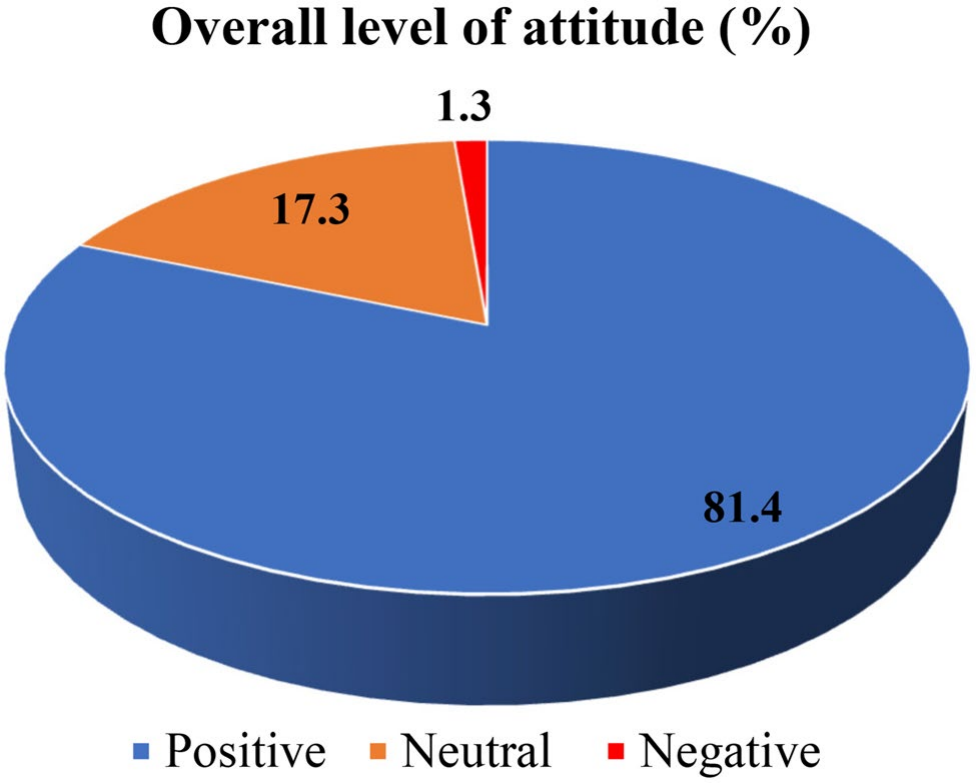
Participants’ over all attitude towards AMR

The majority of the participants 187(81.4%) had a positive attitude towards antimicrobial resistance (Fig. 2).

About 182(79.1%) of respondents agreed that AMR would affect their family’s health, while 189(82.2%) supported more AMR education for final-year students, and 83.5% recognized inappropriate antimicrobial use as a cause of AMR. Additionally, 80.5% linked poor infection control practices to AMR spread, and 188 (81.7%) believed final-year students should receive special training on proper prescribing. The majority 214(93%) agreed to follow hospital antimicrobial guidelines, and 164 (71.3%) recognize AMR as a major global and national problem. However, only 157(68.3%) agreed that socioeconomic status influences antibiotic resistance risk, with 38(16.5%) disagreeing (Table 3).

**Table 3:** Participants’ attitude towards AMR among final year health sciences students at addis Ababa University, 2025

Items	Response		
	Agree *N*(%)	Neutral *N*(%)	Disagree *N*(%)
1. Antimicrobial resistance will affect you and your family’s health.	182 (79.1)	27 (11.7)	21 (9.2)
2. It is necessary to give more education for final year students about antimicrobial resistance.	189 (82.2)	34 (14.8)	7 (3)
3. Inappropriate use of antimicrobials causes antimicrobial resistance.	192 (83.5)	8 (3.5)	30 (13)
4. Poor infection control practices by healthcare professionals will cause the spread of antimicrobial resistance.	185 (80.5)	18 (7.8)	27 (11.7)
5. Final year students should get special training on the appropriate prescribing of antimicrobials before exit.	188 (81.7)	37 (16.1)	5 (2.2)
6. You have to follow the recommendations of your hospital antimicrobial guidelines in the future.	214 (93)	12 (5.2)	4 (1.8)
7. Currently, antimicrobial resistance is a major problem in the world as well as in Ethiopia.	164 (71.3)	(34 14.8)	32 (13.9)
8. People’s socioeconomic status has an effect on the risk of being affected by antibiotic resistance.	157 (68.3)	35 (15.2)	38 (16.5)

### Study participants’ practice towards antimicrobial resistance

**Fig. 3:**
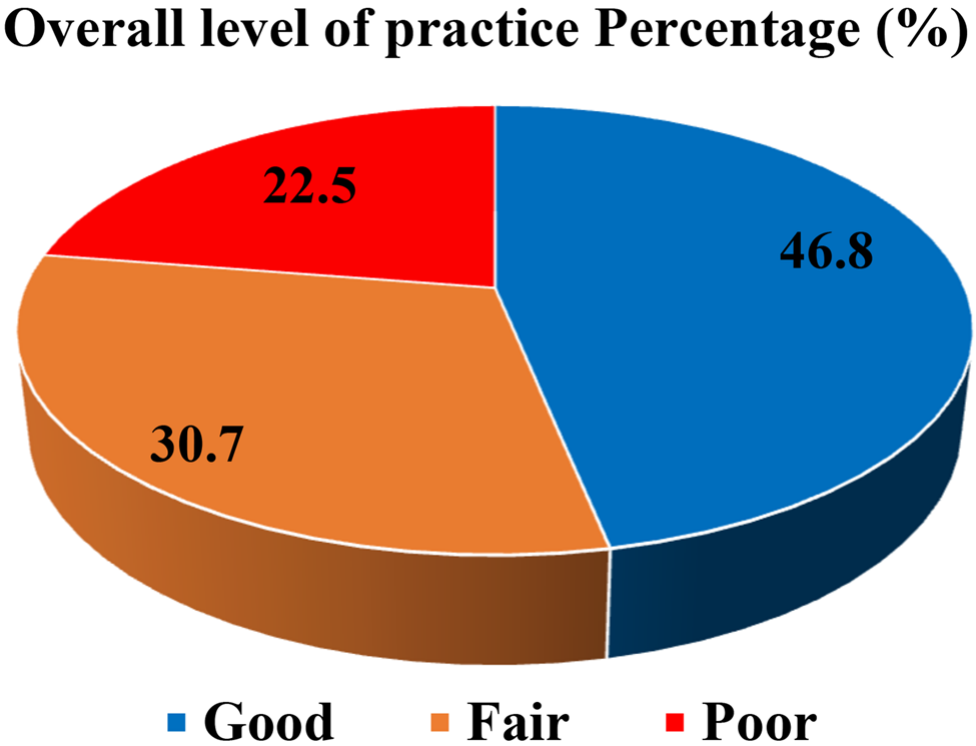
Participants’ over all practice towards AMR

Out of the total respondents, only less than half demonstrated good practice 108(46.8%) followed by fair practice 71(30.7%) (Fig. 3).

A high proportion of the students 194(84.4%) agreed that they consulted a doctor before starting antibiotics. About 98(42.6%) of respondents agreed that they save the remaining antibiotics for the next time they get sick and only 73(31.7%) of the respondents gave the left over antibiotics to their friend/family if they get sick. Only 68 (29.8%) of the respondents stopped taking their antibiotics when they felt better before it is completed the full course antibiotic and 50(21.7%) of the participant would increase the dosage themselves if symptoms didn’t improve. Additionally, 90(39.1%) and 111(48.3%) of the respondent take antibiotics when they have fever and cold respectively (Table 4).

**Table 4:** Participants’ practice towards AMR among final year health sciences students at addis Ababa University, 2025

Items	Response		
	Agree *N*(%)	Neutral *N*(%)	Disagree *N*(%)
1. Do you consult a doctor before starting antibiotics?	194 (84.4)	21 (9.1)	15 (6.5)
2. Do you save the remaining antibiotics for the next time you get sick	98 (42.6)	15 (6.5)	117 (50.9)
3. Do you give the leftover antibiotics to your friend/family if they get sick	73 (31.7)	16 (7%)	141 (61.3)
4. when someone is going to self-medicate with antibiotics, I try to persuade them not to do it	155 (67.4)	40 (17.4)	35 (15.2)
5. If symptoms improve before it is completed the full course antibiotic, you can stop taking it.	68 (29.6)	14 (6)	148 (64.4)
6. Do you take antibiotics when you have a fever	90 (39.1)	18 (7.9)	122 (53)
7. Do you take antibiotics when you have a cold	111 (48.3)	25 (10.9)	94 (40.8)
8. When symptoms don’t improve, will you increase the dosage your self	50 (21.7)	8 (3.5)	172 (74.8)

### Factors associated with study participants’ practice with AMR

For the purpose of data analysis, the three categories of Practice were dichotomized and thereby good and fair practice was taken as optimal, while poor practice as sub-optimal practice.

In the multivariable binary logistic regression model, sex and field of study showed no statistically significant associations with KAP. Participants with moderate and good AMR knowledge had significantly higher odds of optimal practice than those with poor knowledge (AOR = 9.6 and 3.9; *P* = 0.017 and 0.021, respectively). Similarly, a neutral or positive attitude toward AMR significantly increased the likelihood of optimal practice (AOR = 3.71, *P* = 0.021; and AOR = 3.39, *P* = 0.012, respectively (Table 5).

**Table 5:** Factors associated with study participants’ practice with AR among final year health sciences students at addis Ababa University, 2025

Variables	Category	Practice		AOR (95% CI)	P-value
		Optimal N(%)	Sub-optimal N(%)		
Knowledge	Good	42 (21.31)	3(9.01)	9.44(4.12–19.31)	0.017
	Moderate	146 (74.11)	25 (75.76)	3.90(1.07–16.08)	0.021
	Poor	9(4.56)	6 (18.18)	1.00	
Attitude	Positive	151 (84.83)	36(69.23)	3.39(1.82–20.69)	0.012
	Neutral	26 (14.60)	14(26.92)	3.71(1.59–12.60)	0.210
	Negative	1 (1.92)	2 (3.84)	1.00	

## Discussion

In this study, 15(6.5%) of the participants were found to have poor knowledge about AMR. While international comparisons should be made cautiously due to differences in educational contexts and methodologies, this finding appears lower than those from studies conducted in Thailand, Egypt and India, where participants demonstrated a better understanding of antimicrobial resistance [3, 16, 18]. Notably, this figure is also much lower than that reported in a similar study conducted in Ethiopia at the University of Gondar, where 55% of participants exhibited a poor level of knowledge [13]. This discrepancy, particularly with the Gondar study, may be attributed to the difference in enrolled departments and the proportional number of students from each department. In the present study, the majority of the participants were from medicine and pharmacy, in which students are expected to have a better knowledge regarding AMR.

More than 70% of the study participants were well informed about the effect of the frequent use of antibiotics on drug efficacy and antibiotic-induced gut flora disruption, which is high compare to a similar study that reported only 50% of the participants were aware of the issue [13].

Despite this, significant misconceptions were identified that conflict with core antimicrobial stewardship (AMS) principles. For instance, only 64(27.8%) of participants correctly answered whether antibiotics can speed up the recovery of the common cold or flu. This highlights a critical knowledge gap that undermines AMS efforts focused on education about the ineffectiveness of antibiotics for viral infections. While direct comparisons must be made cautiously, this result is significantly lower compared to findings from other studies, where 95% of students in Egypt and 62% of students in India answered this specific question correctly [16, 18]. It is also lower than the result of a local study, where 35% of participants responded correctly. Similarly, a lack of awareness of the systemic nature of AMR was evident, as only 2.6% of study participants emphasized changing prescriber/patient attitudes as AMR control strategies, a cornerstone of AMS. This is also lacking compared to an equivalent study conducted at the University of Gondar which highlighted 51.1% [13].

Furthermore, a clear misunderstanding regarding the indication and effectiveness of antibiotics was observed. Approximately 60% of the study participants believed that antibiotics could kill both viruses and bacteria, a fundamental misconception that counters a key educational objective of AMS programs. This rate is significantly higher compared to a similar study, where only 28% of participants held this misconception. It is important to explicitly state that the validity of such comparisons is limited by participant attributes; the target populations in the Indian [18] and Egyptian [16] studies consisted solely of pharmacy and medical students, while the study conducted at the University of Gondar focused on paramedical students. This might be the possible reason for the disagreement.

Regarding attitude, a substantial percentage (81.4%) of participants held a positive attitude towards AMR and recognized inappropriate use as a cause (82.4%), which is promising. However, the perception of AMR as a major global and local problem (72%) was less prevalent compared to findings from other local and international settings [13].

Interestingly, the majority of participants 189(82.2%) believed that special training on the rational use of antimicrobials and antimicrobial resistance should be provided to final-year students. Based on global standards, like those from the WHO, a stronger desire for more education on AMR and AMS among Ethiopian health professionals highlights a curriculum gap. Integrating a standardized AMS curriculum, reflecting competencies from international frameworks like the one surveyed by Pulcini et al. (2015), is a crucial step for improvement [5]. In the practice section, students demonstrated appropriate practices in certain areas. For instance, 84.4% of respondents reported receiving antimicrobials from doctors, which is an improvement compared to a similar study in China [10]. Additionally, 67% of participants indicated that they discouraged others from self-medicating with antibiotics an encouraging result when compared to a study in Colombia (50%) [21].

However, several self-reported behaviors revealed inappropriate practices that challenge AMS goals. About 49% reported that they would use antimicrobials when experiencing the common cold, a practice directly targeted for reduction by AMS guidelines. Moreover, behaviors that threaten adherence to antibiotic guidelines and encourage improper antibiotic use were noted: approximately 30% of participants stated they would stop taking antibiotics once their symptoms improved, and 42.4% admitted to keeping leftover antibiotics at home for future use. This latter figure is notably higher than that reported by nursing students in China [17].

The difference observed in the research findings may be largely attributed to geographical variation and the specific characteristics of the target population. These practices are likely shaped by more than personal knowledge, reflecting systemic local factors. Weak enforcement of pharmacy regulations prohibiting the illegal over-the-counter sale of antibiotics, coupled with challenges in accessing healthcare, may compel individuals to retain leftover medication for future use or sharing as a pragmatic, though inappropriate solution.

The significant “know-do” gap, evidenced by persistent misconceptions like antibiotics killing viruses, demands immediate, multi-faceted action. Longitudinal curriculum reform integrating AMR/AMS modules with active, case-based learning and communication training **is** recommended. Concurrently, a short-term, pre-deployment AMS training is essential. This feasible intervention could leverage existing internship inductions or Ethiopia’s continuous professional development (CPD) framework [21]. Such targeted training is effective [22] and aligns with Ethiopia’s National Action Plan [23]. Moreover, mandatory student participation in hospital ASPs and stronger EFDA enforcement against illegal over-the-counter antibiotic sales are critical to combat misuse [24, 25].

The limitations of this study include its cross-sectional design, which precludes establishing causal relationships between knowledge, attitudes, and practices. Reliance on self-reported data introduces recall and social desirability biases, potentially reflecting expected rather than actual behaviors. Furthermore, the unequal sample sizes with fewer participants from Midwifery and Medical Laboratory Sciences compared to Medicine limit generalizability across disciplines. Finally, as a single-center study at Addis Ababa University, the findings may not represent the realities of students in other institutions.

## Conclusion

Final-year health sciences students demonstrated overall moderate knowledge and positive attitudes toward AMR, yet specific misconceptions regarding viral infections and suboptimal practices, such as sharing antibiotics, remain prevalent. Notably, the study identified that participants with higher knowledge levels and positive attitudes were significantly more likely to demonstrate optimal practices. These findings suggest that while students possess a foundational understanding, there is a need to further align educational curricula with practical behavioral training. Rather than relying solely on existing regulatory frameworks, a multi-faceted approach that reinforces academic preparation and institutional support is recommended. Specifically, interventions such as short-term, pre-deployment training may serve as a valuable strategy to help address the identified gaps between knowledge and practice among future healthcare professionals.

## Supplementary Information

Below is the link to the electronic supplementary material.

Supplementary Material 1

Supplementary Material 2

## Data Availability

The datasets used and analyzed during the current study are attached as a supplementary data.

## Abbreviations

AMR: Antimicrobial resistance
AMS: Antimicrobial stewardship
CDC: Center for Disease control and prevention
KAP: Knowledge Attitude and Practice
WHO: World Health Organization
SPSS: Statistical Package for Social Sciences
