# Supplementary material for: Knowledge, attitude and practice towards antimicrobial resistance among final-year undergraduate students in the health sciences at Addis Ababa University, Ethiopia
Source: J Pharm Health Care Sci. 2026 Jan 10;12:15. doi: 10.1186/s40780-025-00534-2 (PMC12882412; doi:10.1186/s40780-025-00534-2)
Supplement: Supplementary file 1 — Supplementary Material 1 [file 40780_2025_534_MOESM1_ESM.docx]

## **Appendix-I**

## **Section I: Demographic data**

| Age ___________ |
| --- |
| Sex: _________ |
| Department_______________ |

## **Section II: Questions to assess participants’ Knowledge about antimicrobial resistance**

| R.N | Question (Knowledge) | Options |
| --- | --- | --- |
| 1 | Antibiotic use disturbs gut flora and causes diarrhea or super‑infection | A. Yes |
|  |  | B. No |
| 2 | Antibiotics are powerful medicines to kill viruses and bacteria | A. Yes |
|  |  | B. No |
| 3 | Frequent antibiotic use decreases treatment efficacy | A. Yes |
|  |  | B. No |
| 4 | Antibiotics speed up recovery from cold and cough | A. Yes |
|  |  | B. No |
| 5 | Have you heard about antimicrobial resistance and its consequences | A. Yes |
|  |  | B. No |
| 6 | Which may promote inappropriate antimicrobial use? *(Choose all that apply)* | A. Poor counseling of patients |
|  |  | B. Poor prescriber skills/knowledge |
|  |  | C. Patient self‑medication |
|  |  | D. Inadequate supervision |
| 7 | Which are consequences of antimicrobial overuse? *(Choose all that apply)* | A. Antimicrobial resistance |
|  |  | B. Adverse reactions/medication errors |
| 8 | Which factors may influence starting antimicrobial therapy? *(Choose all that apply)* | A. Patient’s clinical condition |
|  |  | B. Positive microbiology in symptomatic patients |
| 9 | Which promote antimicrobial resistance? *(Choose all that apply)* | A. Inappropriate prescribing habits |
|  |  | B. Lack of diagnostics tools |
|  |  | C. Patient self‑medication without consultation |
|  |  | D. Spread of bacteria in healthcare settings (poor hygiene) |
| 10 | Which support responsible antimicrobial use? *(Choose all that apply)* | A. Consultation with infectious diseases experts |
|  |  | B. Use of local resistance profiles |
|  |  | C. Targeted therapy against likely pathogens |
|  |  | D. Changing attitudes of prescribers and patients to reduce unnecessary use |

##

## **Section III: Questions to assess participants’ attitude about antimicrobial resistance**

| Items | Response | | |
| --- | --- | --- | --- |
|  | Agree | Neutral | Disagree |
| 1. Antimicrobial resistance will affect you and your family’s health. |  |  |  |
| 1. It is necessary to give more education for final year students about antimicrobial resistance. |  |  |  |
| 1. Inappropriate use of antimicrobials causes antimicrobial resistance. |  |  |  |
| 1. Poor infection control practices by healthcare professionals will cause the spread of antimicrobial resistance. |  |  |  |
| 1. Final year students should get special training on the appropriate prescribing of antimicrobials before exit. |  |  |  |
| 1. You have to follow the recommendations of your hospital antimicrobial guidelines in the future. |  |  |  |
| 1. Currently, antimicrobial resistance is a major problem in the world as well as in Ethiopia. |  |  |  |
| 1. People’s socioeconomic status has an effect on the risk of being affected by antibiotic resistance. |  |  |  |

**Section IV. Questions related to practice about antimicrobial use and antimicrobial resistance**

|  | Agree | Neutral | Disagree |
| --- | --- | --- | --- |
| 1. Do you consult a doctor before starting antibiotics? |  |  |  |
| 2 Do you save the remaining antibiotics for the next time you get sick |  |  |  |
| 3. Do you give the leftover antibiotics to your friend/family if they get sick |  |  |  |
| 4. You have the same symptoms before, so you buy the antibiotics or ask your doctor them |  |  |  |
| 5. If symptoms improve before it is completed the full course antibiotic, you can stop taking it |  |  |  |
| 6. Do you take antibiotics when you have a fever |  |  |  |
| 7. Do you take antibiotics when you have a cold |  |  |  |
| 8. When symptoms don’t improve, will you increase the dosage your self |  |  |  |
